# The role of m6A-related genes in the prognosis and immune microenvironment of pancreatic adenocarcinoma

**DOI:** 10.7717/peerj.9602

**Published:** 2020-09-28

**Authors:** Rong Tang, Yiyin Zhang, Chen Liang, Jin Xu, Qingcai Meng, Jie Hua, Jiang Liu, Bo Zhang, Xianjun Yu, Si Shi

**Affiliations:** Department of Pancreatic Surgery, Fudan University Shanghai Cancer Center, Shanghai, China; Department of Oncology, Shanghai Medical College, Fudan University, Shanghai, China; Shanghai Pancreatic Cancer Institute, Shanghai, China; Pancreatic Cancer Institute, Fudan University, Shanghai, China

**Keywords:** Pancreatic adenocarcinoma, RNA methylation, m6A, Prognosis, Immunity, Gemcitabine resistance

## Abstract

**Background:**

Pancreatic adenocarcinoma (PAAD) is among the most lethal diseases and has a dismal prognosis; however, efficient treatment is currently limited. Several studies have observed epigenetic variation during tumorigenesis, suggesting the potential role of RNA methylation, especially N6-methyladenosine (m6A) modification, as a novel epigenetic modification mediating PAAD prognosis.

**Methods:**

The expression levels of m6A-related genes were downloaded from The Cancer Genome Atlas-Pancreatic Adenocarcinoma (TCGA) and Genotype-Tissue Expression (GTEx) projects, and the findings were validated in four Expression Omnibus (GEO) datasets. A predictive model was constructed using a lasso regression and evaluated by a survival analysis and receiver operating characteristic curve. Consensus clustering identified two distinct subgroups with different immune activity signatures based on the expression pattern of m6A-related genes. The relationship between the mutation state of m6A-related genes and infiltration of immune cells was established and visualized using Tumor Immune Estimation Resource (https://cistrome.shinyapps.io/timer/).

**Results:**

Fourteen of twenty-one m6A-related genes were differentially expressed between PAAD and normal tissues in TCGA-GTEx cohort. Among these genes, *HNRNPC, IGF2BP2* and *YTHDF1* were further validated in four GEO datasets. Moreover, an m6A-based model exhibited moderate accuracy in predicting overall survival in PAAD samples. Additionally, potential m6A modification targets were screened by selecting genes from a set of 23,391 genes that not only harbored the most m6A-modified sites but also showed a robust correlation with PAAD survival. Moreover, we correlated the expression level of m6A-related genes with the immune microenvironment of pancreatic cancer for the first time. Specifically, both arm-level gain and deletion of *ALKBH5* decreased the infiltration of CD8+T cells (*P* < 0.05 and *P* < 0.01, respectively).

**Conclusion:**

Collectively, our findings suggest a novel anticancer strategy for restoring balanced RNA methylation in tumor cells and guide clinical physicians in developing a new practical approach for considering the impact of related genes on prognosis.

## Introduction

Pancreatic adenocarcinoma (PAAD) is among the most lethal diseases and has a dismal prognosis (Moore & Donahue, 2019). Worldwide, the health burden of PAAD, which causes 331,000 deaths per year according to GLOBOCAN 2012 estimates, is annually increasing (Yadav & Lowenfels, 2013). Many factors, such as its robust chemoresistance, lead to unfavorable survival parameters; however, efficient treatment methods remain limited (Amrutkar & Gladhaug, 2017; Balachandran, Beatty & Dougan, 2019; Zeng et al., 2019). Hence, elucidating the mechanism underlying pancreatic tumorigenesis is urgently needed.

In recent decades, advances in epigenetic techniques have provided scientists with new insight into PAAD (Mardis, 2008; Shen et al., 2019). Several studies uncovered a series of epigenetic variations in DNA and histone methylation during PAAD tumorigenesis (Bailey et al., 2016; Kisiel et al., 2015; Li et al., 2018; Neureiter et al., 2014), and the findings demonstrate promising clinical value (Majumder et al., 2019; Matsubayashi et al., 2006). However, as a novel epigenetic modification, the role of RNA methylation in cancers, especially pancreatic neoplasms, is not well established. Only a few publications investigating the relationship between RNA methylation modification and PAAD are available in the literature (Abukiwan et al., 2019; He et al., 2018). N6-methyladenosine (m6A) is among the most common RNA modifications in eukaryotes and regulates RNA behaviors, such as splicing and protein-coding ability (Dai et al., 2018; Wang et al., 2014). Dysregulated m6A modifications in the transcripts of some oncogenes (such as *Snail*) or tumor suppressors (such as *PHLPP2*) are involved in tumor proliferation and metastasis (Lin et al., 2019; Liu & Eckert, 2018). The installation, recognition, and removal of m6A marks are performed by specific molecules called “writers”, “readers”, and “erasers”, respectively (Liu et al., 2017; Meyer & Jaffrey, 2014). Hence, the ectopic expression of these m6A-related genes may affect the prognosis of PAAD through the regulation of the m6A modification of key genes in tumor progression. In addition, a recent study reported that a key m6A reader, i.e., *YTHDF1*, undermines the durable neoantigen-specific immunity by interacting with transcripts encoding lysosomal proteases, suggesting that altered m6A modification could facilitate tumor cells to escape capture from immune cells. Determining whether m6A influences the prognosis and immune microenvironment of PAAD is important not only for further understanding the epigenetic changes occurring during pancreatic tumorigenesis but also for the accurate identification of potential novel targets for early diagnosis and efficient treatment.

In this context, this study was designed to compare the expression levels of m6A-related genes in PAAD samples with those in matched normal pancreatic tissue and further investigate the role of these genes in the survival and immune microenvironment of pancreatic cancer.

## Methods

### Comparison of the expression levels of m6A-related genes between PAAD and normal pancreatic tissues

Genes closely associated with the installation, recognition and removal of m6A marks were identified in the literature and analyzed in the present study. As the main cohort, this study incorporated transcriptome data of PAAD and normal pancreatic tissue samples originally available in the TCGA-PAAD and GTEx projects and further analyzed the data via Gene Expression Profiling Interactive Analysis (GEPIA) (http://gepia.cancer-pku.cn) (Li et al., 2017; Tang et al., 2017), which is an online platform, to visualize the TCGA-PAAD and GTEx data. The validation cohort consisted of the following two parts: 1. transcriptome data of PAAD and matched normal tissues collected from the GEO database for validation at the transcriptome level and 2. immunohistochemical data collected from the Human Protein Atlas (https://www.proteinatlas.org/) for validation at the protein level.

An ANOVA was used to compare the expression levels of m6A-related genes in GEPIA with those in the TCGA and GTEx data. GEO2R, which is a built-in tool in the GEO database, was used to validate the differential expression of the m6A-related genes based on the GEO datasets.

### Construction of a prognostic gene model

The clinical data of the main cohort included the survival time, survival status, TNM classification, stage, age and sex and were downloaded from TCGA-PAAD. First, we applied a univariate Cox regression analysis to identify the m6A-related genes significantly associated with the prognosis of PAAD (*P* < 0.05). Then, a lasso regression was conducted to calculate the risk coefficient of each gene after the removal of some genes with a risk of overfitting according to the partial likelihood deviance and lambda value (the lambda value is determined by the smallest likelihood deviance; the coefficient-lambda curve shows the genes that are eligible when the lambda value is determined). The lasso risk was calculated using the following formula: lasso risk = ${\mathop{\sum }\nolimits }_{i=1}^{n}Coef\times xi$. Finally, the remaining genes were utilized to construct a predictive model of the prognosis of PAAD. The samples with a top 50% risk value were regarded as “high risk”, while the samples with a bottom “50%” risk value were considered “low risk”. A ROC curve was generated to assess the predictive value of the constructed model. Univariate and multivariate Cox analyses were performed to identify the independent prognostic factors of PAAD (*P* < 0.05). A validation cohort was established from the GEO dataset and used to confirm the accuracy of the prognostic model based on the TCGA cohort. We adjusted the expression level of each m6A-related gene in the GEO dataset due to the use of different sequencing platforms to evaluate the accuracy of our prognosis model, which ensured optimized comparability between the validation cohort and TCGA cohort. First, we standardized each gene’s expression level using the following formula: ${x}_{std}= \frac{{x}_{i}-\overline{x}}{s} $, $\overline{x}$ = $ \frac{1}{n} {\mathop{\sum }\nolimits }_{i=1}^{n}{x}_{i}$, s = $\sqrt{ \frac{1}{n-1} {\mathop{\sum }\nolimits }_{i=1}^{n}({x}_{i}-\overline{x})^{2}}$; Then, we adjusted each *X*_*std*_ to match the training data of TCGA using the following formula: ${x}_{adj}={x}_{std}\times {s}_{train}+{\overline{x}}_{train}$. A survival analysis was performed using the R package “survival”. The “glmnet” package was used to perform a Cox proportional hazards regression analysis with least absolute shrinkage (glmnet, version 2.0-18). Univariate and multivariate Cox regression analyses were performed to provide evidence that the prognostic gene model was independent of other clinicopathological factors. A lasso regression was conducted using the packages “glmnet” and “survival”. The package “survivalROC” was used to depict the ROC curve. The risk score plot was generated by the package “pheatmap”. *P* < 0.05 was considered statistically significant.

### Exploration of potential m6A modification targets

Initially, we acquired the top 100 genes that harbored the most m6A-modified sites from among 23,391 genes in RMBase (Xuan et al., 2018) and then plotted the survival curve to evaluate the role of these genes in PAAD prognosis (Zhu et al., 2019). Additionally, we screened the top 20 genes that were closely associated with PAAD survival and then determined the number of m6A-modified sites in their transcriptional products. The mutation information of the m6A-related genes was obtained and visualized in cBioPortal (http://www.cbioportal.org/) (Cerami et al., 2012; Gao et al., 2013). The survival analysis compared the overall survival time and progression-free time between patients with mutated m6A-related genes and those without.

### Investigation of the role of m6A-related genes in the immune microenvironment of pancreatic cancer

According to the similarities in the gene expression levels, the PAAD samples in the TCGA dataset were clustered into different groups using “ConsensusClusterPlus” (50 iterations, resample rate of 80%, and Pearson correlation; http://www.bioconductor.org/). We performed a PCA via an R package in R v3.6.1 to study the gene expression patterns in different PAAD groups. A single sample gene-set enrichment analysis (ssGSEA) was conducted based on the expression level of 29 immunity-associated signatures using the R package “GSEAbase”. The activity of immune signatures and the immune score were compared between two subgroups using a *t*-test. The tumor purity of the two subgroups was compared using a Wilcox test. The package “Estimate” was used to calculate the immune score, stroma score and tumor purity of each tumor sample. The relationship between the mutation state or expression level of m6A-related genes and infiltration of immune cells was established and visualized using Tumor Immune Estimation Resource (https://cistrome.shinyapps.io/timer/).

We selected the gene that showed the highest hazard ratio with PAAD prognosis and analyzed its role in the alteration of immune signatures. Quartiles were used to assess the expression level of this gene. The samples with the top 25% expression level of this gene are regarded as “high expression”, while the samples with the bottom 25% expression level of this gene are classified as “low expression”.

## Results

### Differential expression of m6A-related genes between tumor and normal samples

**Figure 1:**
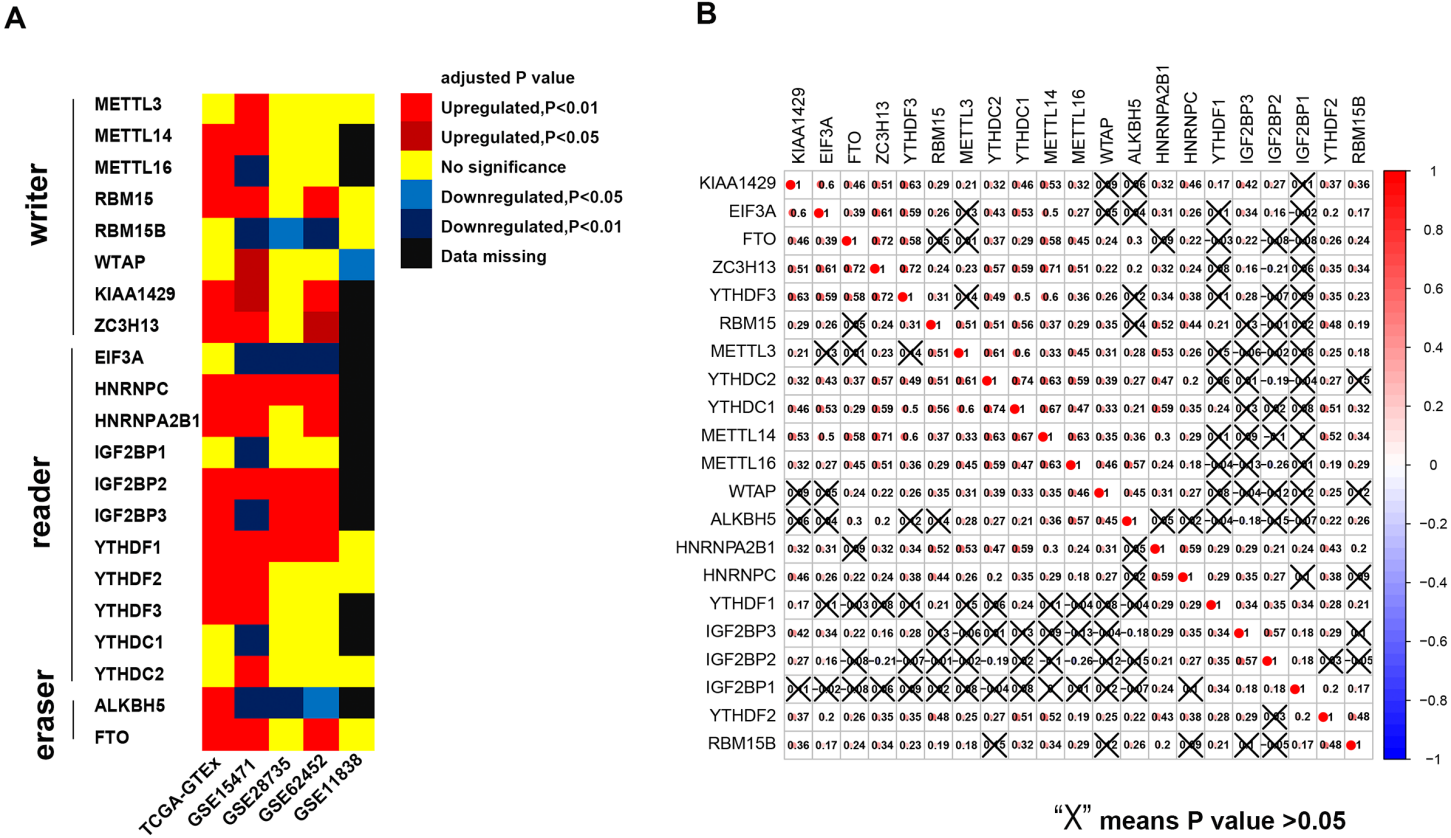
Expression pattern of m6A-related genes and co-expression analysis of 21 m6A-related genes. (A) HNRNPC, IGF2BP2 and YTHDF2 are constantly over-expressed in PAAD in the TCGA-GTEx cohort and at least three of the four GEO datasets. (B) Broad co-expression network exists among the 21 m6A-related genes. The cross-out cell indicates that the co-expression correlation between two genes is not significant (*P* < 0.05).

The results of this study are summarized in a flow chart (Fig. S1). We selected 21 genes (*METTL3, METTL14, METTL16, WTAP, KIAA1429, EIF3, IGF2BP1-3, RBM15, RBM15B, ZC3H13, YTHDC1, YTHDC2, YTHDF1, YTHDF2, YTHDF3, HNRNPC, HNRNPA2B1, FTO* and *ALKBH*) based on the existing literature (Batista, 2017; Chai et al., 2019; Dai et al., 2018). The translated products of these genes are essential for the installation, removal and recognition of m6A marks (Table 1). We identified 14 differential m6A-related genes in the TCGA-GTEx cohort. Specifically, five m6A writers (*METTL14, METTL16, KIAA1429, RBM15* and *ZC3H13*) were upregulated in PAAD, and seven m6A readers (*YTHDF1, YTHDF2, YTHDF3, IGF2BP2, IGF2BP3, HNRNPA2B1* and *HNRNPC*) were highly expressed in PAAD. Similarly, two major RNA demethylases, i.e., *FTO* and *ALKBH5*, were overexpressed in the tumor samples. In addition, four Gene Expression Omnibus (GEO) datasets (GSE15471, GSE28735, GSE62452 and GSE11838) were used as validation cohorts (Table S1). Genes with the same expression pattern in the TCGA-GTEx cohort and more than three of the four GEO datasets are defined as “validated genes”. The results revealed that *HNRNPC*, *IGF2BP2* and *YTHDF1* are highly expressed in PAAD with powerful evidence, while *METTL3* and *YTHDC2* are less likely to have differential expression (Fig. 1A).

**Table 1:** The basic information of the included m6A-related genes.

Gene_name	The role in m6A^a^	Ensemble	Location
METTL3	Writer	ENSG00000165819	Chromosome 14, NC_000014.9
METTL14	Writer	ENSG00000145388	Chromosome 4, NC_000004.12
METTL16	Writer	ENSG00000127804	Chromosome 17, NC_000017.11
WTAP	Writer	ENSG00000146457	Chromosome 6, NC_000006.12
KIAA1429	Writer	ENSG00000164944	Chromosome 8, NC_000008.11
RBM15	Writer	ENSG00000162775	Chromosome 1, NC_000001.11
RBM15B	Writer	ENSG00000259956	Chromosome 3, NC_000003.12
ZC3H13	Writer	ENSG00000123200	Chromosome 13, NC_000013.11
EIF3A	Reader	ENSG00000107581	Chromosome 10, NC_000010.11
IGF2BP1	Reader	ENSG00000159217	Chromosome 17, NC_000017.11
IGF2BP2	Reader	ENSG00000073792	Chromosome 3, NC_000003.12
IGF2BP3	Reader	ENSG00000016797	Chromosome 7, NC_000007.14
YTHDC1	Reader	ENSG00000083896	Chromosome 4, NC_000004.12
YTHDC2	Reader	ENSG00000047188	Chromosome 5, NC_000005.10
YTHDF1	Reader	ENSG00000149658	Chromosome 20, NC_000020.11
YTHDF2	Reader	ENSG00000198492	Chromosome 1, NC_000001.11
YTHDF3	Reader	ENSG00000185728	Chromosome 8, NC_000008.11
HNRNPC	Reader	ENSG00000092199	Chromosome 14, NC_000014.9
HNRNPA2B1	Reader	ENSG00000122566	Chromosome 7, NC_000007.14
FTO	Eraser	ENSG00000140718	Chromosome 16, NC_000016.10
ALKBH5	Eraser	ENSG00000091542	Chromosome 17, NC_000017.11

**Notes.**

a m6A, N6-methyladenosine

In addition, we used immunohistochemical data from the Human Protein Atlas to evaluate the expression of the m6A-related genes at the protein level (Table S1), although data for some m6A-related genes were unavailable on the website. The results showed that the protein expression of *RBM15* is upregulated in pancreatic cancer, which is similar to the results of the TCGA-GTEx cohort and two GEO datasets.

Pearson coefficients were used to quantify the magnitude of the co-expression of the m6A-related genes. Ten gene pairs were found to be highly co-expressed with a Pearson coefficient greater than 0.6 (Fig. 1B). Among these pairs, the *YTHDC1-YTHDC2* pair had the strongest correlation magnitude (0.74); however, *YTHDF1* and *IGF2BP1-3* exhibit a lower correlation with the other m6A-related genes.

### Prognostic model based on three m6A-related genes can predict the survival of patients with PAAD with intermediate accuracy

**Figure 2:**
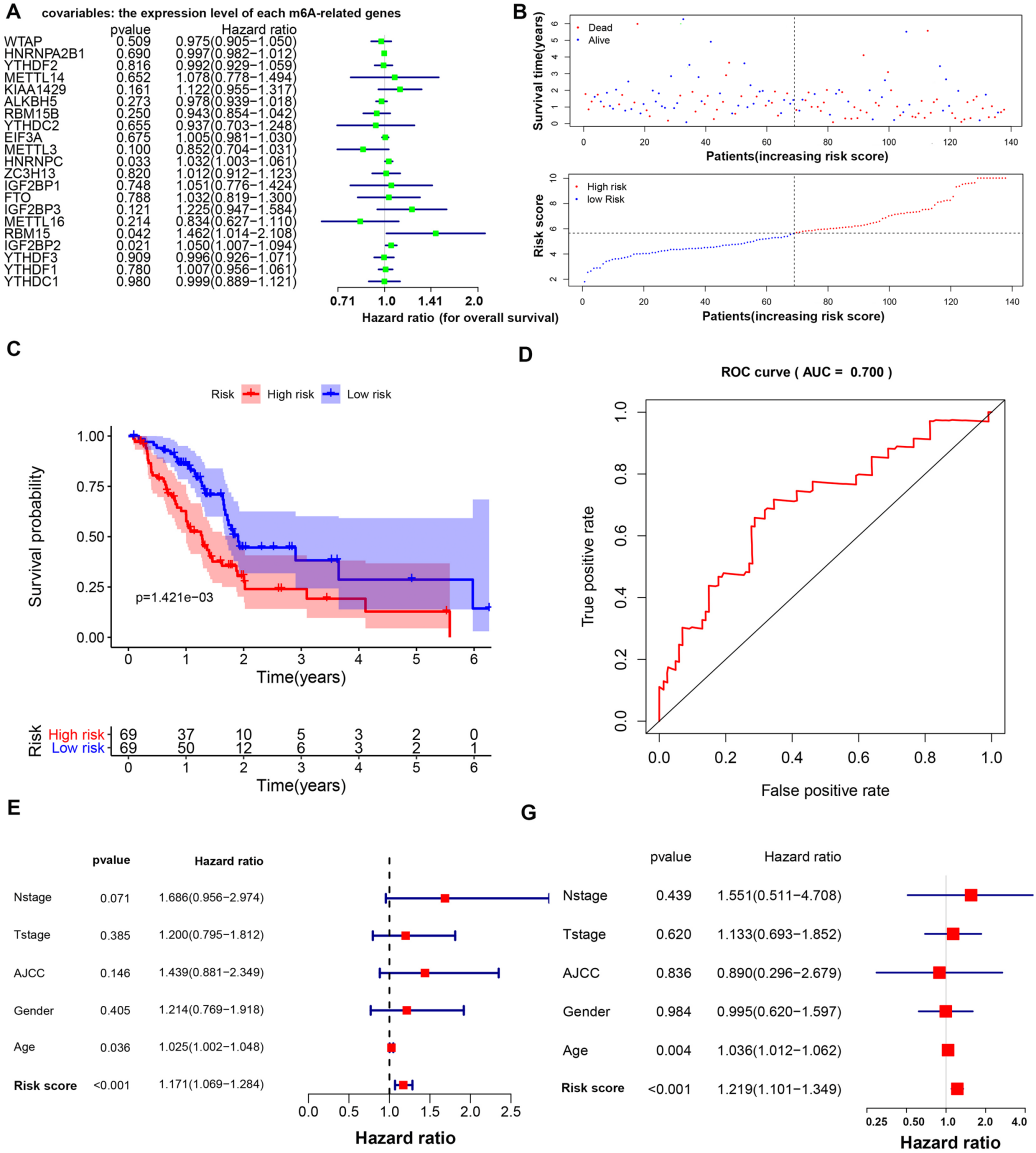
Prognostic model based on m6A-related genes with moderate accuracy in predicting overall survival in PAAD. (A) Univariate Cox analysis showing the hazard ratio of each m6A-related gene in predicting overall survival in PAAD. (B) Risk score curve profiling the survival situation of patients with risk scores calculated by the present model. (C) Survival analysis of the high- and low-risk PAAD groups, which were classified according to the expression levels of m6A-related genes. (D) ROC curve showing the moderate accuracy of the constructed prognostic model of PAAD. (E–F) Univariate and multivariate Cox analyses of clinical parameters and lasso risk for overall survival in PAAD. The covariables are the N stage, T stage, AJCC (American Joint Committee on Cancer) stage, gender and sex of the PAAD patients. The response variable is the overall survival time of the patients.

Three m6A-related genes significantly associated with the prognosis of PAAD were screened through a univariate Cox analysis (Fig. 2A). To avoid the overfitting phenomenon in the subsequent model construction, a least absolute shrinkage and selection operator (LASSO) regression was used to detect whether dimensionality reduction is possible by eliminating redundant genes. According to the partial likelihood deviance and lambda values, all three genes are suggested to be included in the model construction (Figs. S2A to S2C). The lasso regression provided a risk score to each sample based on the expression level of the three included genes. The samples were divided into two groups in a dichotomous fashion according to the ranking of the risk score (Fig. 2B). Overall, the group of patients with the low lasso risk score showed a longer survival time than the group of patients with a high lasso risk score (*P* < 0.005) (Fig. 2C). Then, a receiver operating characteristic (ROC) curve was generated to assess the predictive accuracy of this model (Fig. 2D). The lasso risk score was obtained as follows: lasso risk score = (0.2623*expression level of RBM15) + (0.0168*expression level of HNRNPC) + (0.0367*expression level of IGF2BP2). The model exhibited intermediate accuracy with an area under the ROC curve (AUC) of 0.700. One GEO dataset (GSE71729) comprising 125 pancreatic ductal adenocarcinoma (PDAC) samples was used as a validation cohort to verify the accuracy of this prognostic model. Fifty-five samples were considered high-risk, and the other 70 samples were considered low-risk based on the coefficient calculated by the TCGA-based model (Fig. S3A). Similarly, the samples with low risk scores showed longer overall survival (OS) times than those with high risk scores (*P* < 0.05), supporting the accuracy and practicability of this model (Fig. S3B).

### Risk score based on three m6A-related genes is a reliable indicator of a shorter OS

The age and lasso risk score were significantly associated with a worse prognosis of PAAD in both the univariate and multivariate Cox regression analyses, suggesting that these factors are independent prognostic factors. Interestingly, the grade, stage, T stage, N stage and sex were unrelated to the survival of patients with PAAD in this study (Figs. 2E to 2F).

### Potential m6A modification targets and summary of the mutation information of the m6A-related genes

A survival analysis was performed with the top 100 genes that harbored the most m6A-modified loci, which ranged from 116 to 518 in number (Table S3). Sixteen of these genes were found to be strongly associated with the prognosis of PAAD (*P* < 0.05) (Fig. 4). Additionally, we determined the number of m6A-modified sites in the top 20 genes that were closely associated with PAAD survival (Table S4) and found that 17 of the 20 genes harbored between five and 38 m6A-modified loci.

Four cohorts (ICGC, QCMG, TCGA and UTSW) were included in this study to analyze the mutation information of the m6A-related genes. Overall, these genes were mutated in 118 samples (118/848) in which deletion mutations occurred mainly in *METTL14, WTAP, YTHDC1* and *YTHDF2*, whereas amplification mutations occurred mostly in *KIAA1429* and *YTHDF1* (Fig. S5). The patients with samples that did not exhibit mutations in m6A-related genes showed longer OS and disease/progression-free survival times than those with genetic alterations by a Kaplan–Meier analysis (Figs. S6A to S6B). Among the genes with mutations crucial for PAAD tumorigenesis, *ZNF814* was frequently concurrently mutated with the m6A-related genes (Fig. S6C). We further compared the copy number alteration frequency of four driver genes between the samples with and those without mutations of the m6A-related genes. The results showed that the copy number alteration frequency of *TP53* was significantly increased in the samples with mutations in the m6A-related genes, suggesting that *TP53* may be involved in m6A regulation in PAAD (Figs. 6C to 6D).

### Subgroup of PAAD with a specific expression pattern of m6A-related genes exhibits different immune signatures

**Figure 3:**
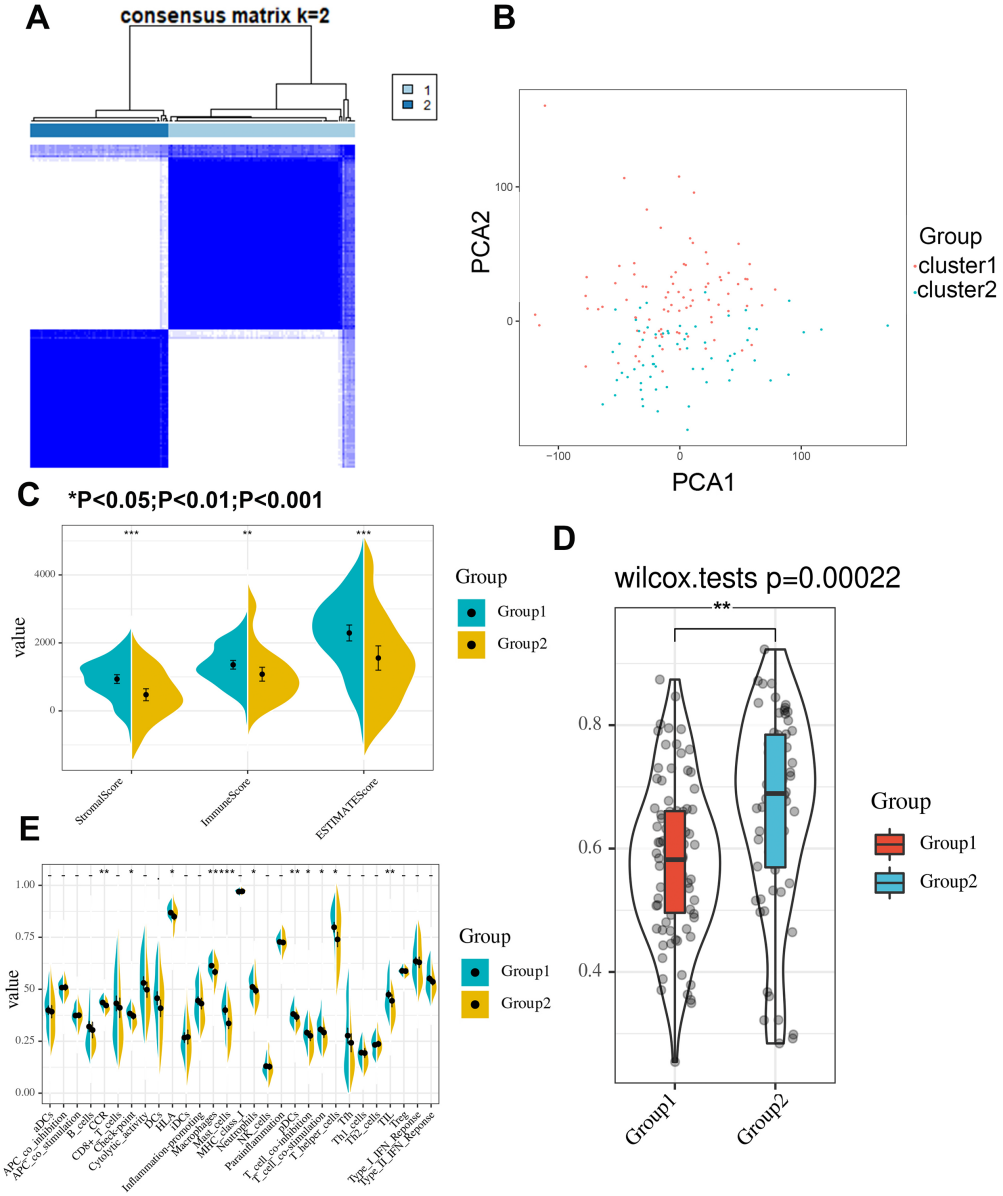
Consensus clustering identifies two subgroups with distinct immune scores, stromal scores, tumor purities and immune signatures. (A) Consensus clustering identifies two subgroups based on the expression level of m6A-related genes. (B) Principle component analysis confirmed the independence between the two subgroups. (C–D) The stromal score, immune score and tumor purity significantly differ between the two clustered groups. (E) The cytotoxic response, checkpoint, HLA, macrophage, mast cell, neutrophil, dendritic cell, T cell co-stimulation/inhibition, T helper cell and tumor infiltrating lymphocyte signatures significantly differ between the two clustered groups.

A clustering analysis based on the expression levels of the m6A-related genes in the PAAD samples was performed, and an increasing trend of the cumulative distribution function (CDF) value with respect to the consensus index was considered indicative of an appropriate classification (Figs. S7A to S7B). Finally, two subgroups with similar sample sizes were clearly classified (Fig. 3A). We further confirmed the independence of the two subgroups by a principal component analysis (PCA), which further supported the clustering results (Fig. 3B). We also evaluated the difference between the two subgroups in terms of the stromal score, immune score, immune signatures and tumor purity (Fig. 3C). The results demonstrated that group 1 had higher stromal and immune scores but a lower tumor purity (Fig. 3D). Moreover, group 1 exhibited a better cytotoxic response and more check point signatures, HLA, macrophages, mast cells, neutrophils, etc., suggesting that the difference in the expression pattern of m6A-related genes leads to an altered immune microenvironment (Fig. 3E).

### Expression level and mutation state of m6A-related genes in the immune microenvironment of pancreatic cancer

**Figure 4:**
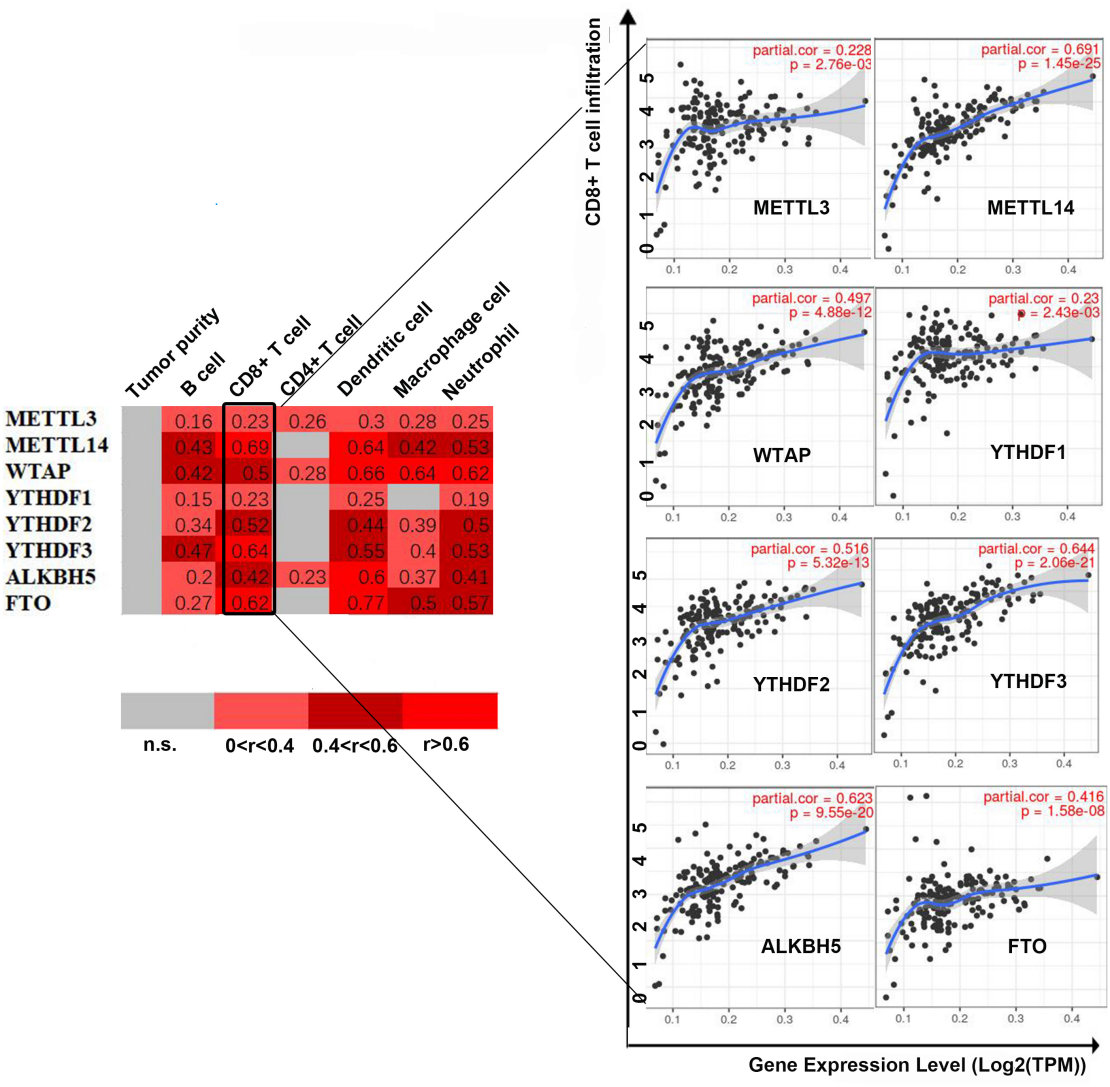
Correlation between infiltrating immune cells and the expression level of m6A-related genes. The correlation coefficient is labeled “r”. *r* > 0 indicates that the number of infiltrating immune cells is positively correlated with the gene expression level. We further use color intensity to reflect the correlation strength (0 0.6: level 3).

**Figure 5:**
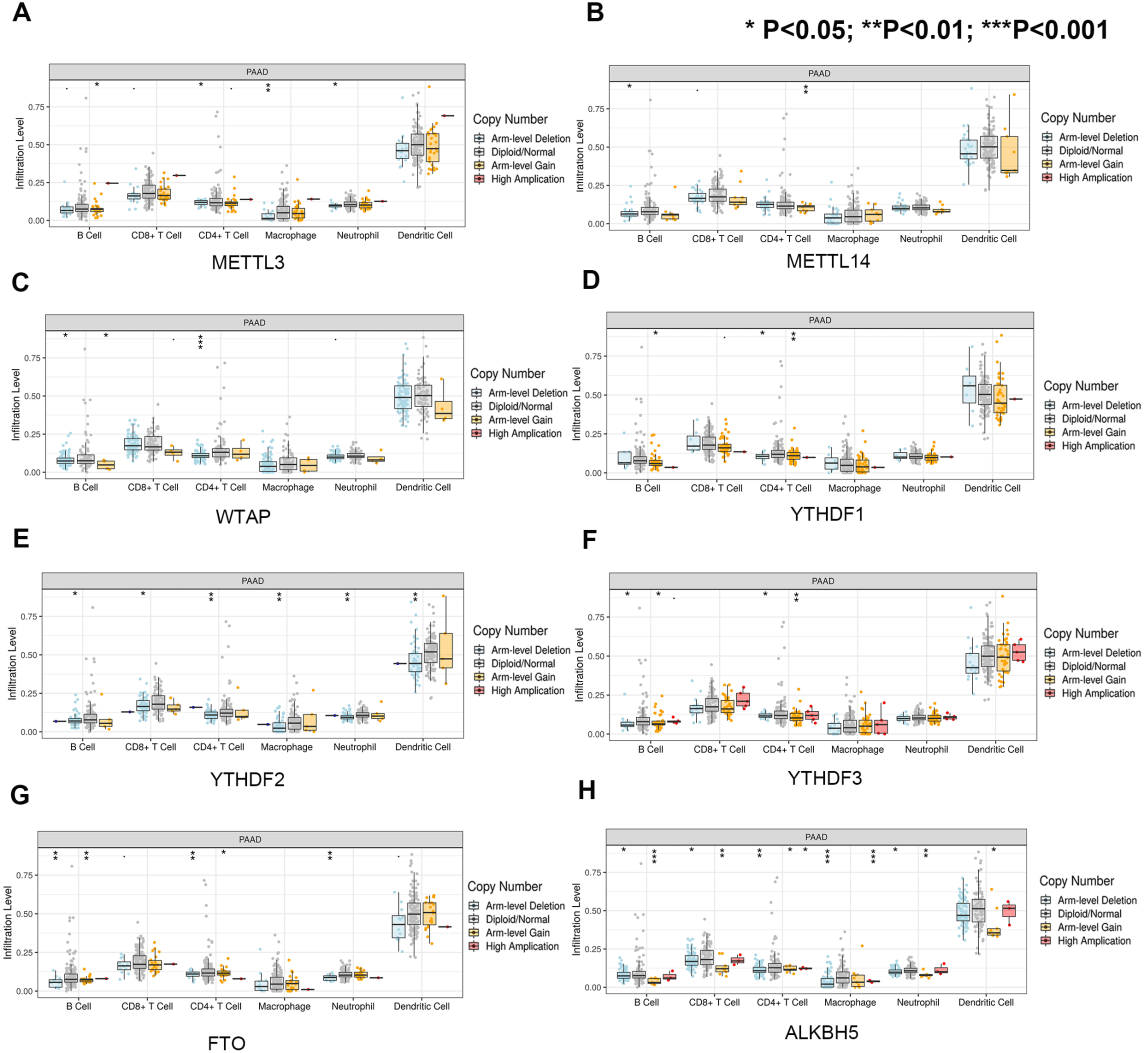
Univariate and multivariate Cox analyses of clinical parameters and the lasso risk for overall survival in PAAD. (A) The association between copy-number variation of METTL3 and infiltrated immune cells. (B) The association between copy-number variation of METTL14 and infiltrated immune cells. (C) The association between copy-number variation of WTAP and infiltrated immune cells. (D) The association between copy-number variation of YTHDF1 and infiltrated immune cells. (E) The association between copy-number variation of YTHDF1 and infiltrated immune cells. (F) The association between copy-number variation of YTHDF3 and infiltrated immune cells. (G) The association between copy-number variation of FTO and infiltrated immune cells. (H) The association between copy-number variation of ALKBH5 and infiltrated immune cells. Each dot provides a comparison of the tumor infiltration levels among tumors with different somatic copy number alterations of a given gene. Box plots are presented to show the distributions of each immune subset at each copy number status in selected cancer types. The infiltration level in each SCNA category is compared with the normal level using a two-sided Wilcoxon rank-sum test. Arm-level deletion/gain indicates that the alteration is only limited to the chromosome arm. High amplification/deep deletion indicates that the alteration exists at the chromosome level.

**Figure 6:**
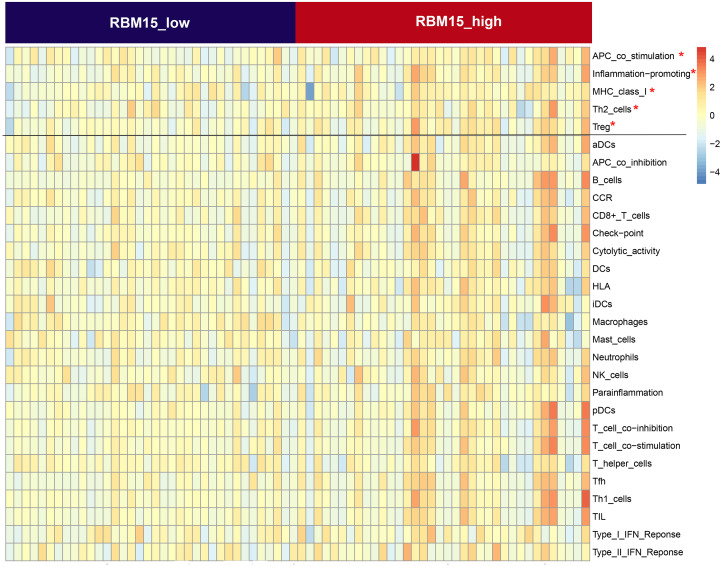
Comparison of the groups with high and low RBM15 expression in terms of 29 immune signatures. *Z*-score reflects the relative activity of immune signatures; red indicates high activity of a specific immune signature, and blue indicates low activity of a specific immune signature.

First, we correlated the expression of the eight most studied m6A-related genes with immune cells in pancreatic cancer (Fig. 4). All eight genes were positively associated with the number of CD8+ T cells after adjusting for the tumor purity, suggesting that m6A modification potentially regulates CD8+T cell aggregation. However, the presence of arm-level deletion or gain mutations was negatively correlated with the CD8+T cell number (Fig. 5). Specifically, both the arm-level gain and deletion of *ALKBH5* decreased the infiltration of CD8+T cells (*P* < 0.05 and *P* < 0.01, respectively). *RBM15* was the gene with the highest hazard ratio to PDAC survival; hence, we further analyzed the correlation between *RBM15* and 29 immune signatures. The results showed that a higher expression of *RBM15* is positively associated with APC co-stimulation, inflammation promotion, MHC class I, Th2 cells and Treg cells (Fig. 6).

## Discussion

Our study revealed the differential expression of m6A-related genes between PAAD and normal pancreatic tissue based on combined TCGA-PAAD and GTEx datasets. Furthermore, we constructed a prognostic model to predict the survival of patients with PAAD with intermediate accuracy. In addition, we explored the potential relationship between the expression level and mutation state of m6A-related genes and the immune microenvironment in pancreatic cancer. The findings implied that disruption of the RNA methylation system may play a crucial role in the progression of PAAD.

Epigenetics focuses on inheritable gene expression alterations independent of the primary DNA sequence, including DNA methylation, genomic imprinting, RNA editing, etc. Accumulating evidence has shown that epigenetic alteration is closely associated with the pathophysiology occurring during PAAD progression, and oncogenic mechanisms beyond DNA mutations have been accepted and emphasized by most scientists. The methylation level of long interspersed nucleotide element-1 (*LINE-1*) is a good surrogate marker of the global DNA methylation level. Yamamura et al. (2017) observed that the level of *LINE-1* methylation in PAAD samples is significantly lower than that in normal tissues, suggesting that global DNA methylation is downregulated in PAAD. Additionally, Koutsioumpa and colleagues found that histone lysine (K)-specific methyltransferase 2D (*KMT2D*) is transcriptionally inhibited in human pancreatic tumors through DNA methylation, which hinders the process of histone methylation and further promotes tumor growth. In addition, the results of some translational studies in the literature have improved the diagnosis of PAAD. A recent meta-analysis involving 1,243 patients found that hypermethylation of cell-free DNA is correlated with worse survival outcomes in PAAD patients (Chen et al., 2018a). In addition, Kisiel et al. (2015) detected 19 DNA methylation markers in pancreatic juice that exhibited promising diagnostic accuracy in predicting PAAD. In conclusion, understanding epigenetic alterations is crucial for improving the understanding of PAAD considering the aforementioned findings and facilitating access to its early diagnosis and efficient treatment.

RNA methylation was established as a novel component of epigenetics in the 1970s and has gradually become a popular research area in recent years (Kontur & Giraldez, 2017; Sergiev et al., 2018; Stojkovic & Fujimori, 2017). Many studies have investigated whether methylation modification of specific RNAs is involved in tumorigenesis (Boriack-Sjodin, Ribich & Copeland, 2018; Thapar et al., 2019; Tuncel & Kalkan, 2019). For example, Chen et al. observed an elevated expression of *METTL3* in hepatocellular carcinoma. Further biochemical experiments have shown that a high expression of *METTL3* induces increased m6A modification of the tumor suppressor *SOCS2*. Then, *YTHDF2*, which is an m6A reader, directly recognizes m6A-modified *SOCS2* mRNA and subsequently induces the degradation of *SOCS2* (Chen et al., 2018b). In contrast, the abundance of m6A detected in approximately 70% of endometrial tumors was lower than that detected in the normal endometrium. Subsequent experiments implied that the decline in the m6A level was likely due to either a *METTL14* R298P mutation or the reduced expression of *METTL3* (Liu et al., 2018).

Regarding PAAD, a recent study demonstrated that *YTHDF2* is upregulated in PAAD at both the mRNA and protein levels. Highly expressed *YTHDF2* promotes tumor proliferation by activating the *AKT/GSK3 β/cyclin D1* pathway and suppresses migration and invasion by binding m6A sites on and decomposing yes-associated protein (*YAP*) mRNA (Chen et al., 2017). Our results partially confirm this finding by showing that the expression of *YTHDF2* is highly elevated in the PAAD samples in the TCGA-GTEx cohort and a GEO dataset (GSE15471) (*P* < 0.001). However, we did not find a correlation between *YTHDF2* and shorter OS times in PAAD patients (Fig. 2A). Given that *YTHDF2* facilitates tumor proliferation but prohibits migration, we compared the level of *YTHDF2* in tumor tissues in different T and N stages. Unfortunately, we did not find a correlation between the *YTHDF2* level and either the T or N stage via a *t*-test or a one-way analysis of variance (ANOVA) (*P* = 0.425 and 0.323, respectively) (Fig. S8). A possible explanation for this inconsistency is the lack of representativeness of cell-level experiments compared with that of sequencing data obtained directly from human samples. In addition, the number of T1 and T4 samples was extremely limited, which may have introduced unexpected bias to our study. Another study reported that the miR-17-5p methylation level in serum samples can distinguish patients with early PAAD from healthy controls with extremely high sensitivity and specificity (Konno et al., 2019) and, thus, appears promising for development as a convenient method for the detection of PAAD. Therefore, in addition to targeting mRNA for modification, m6A-related genes could act on other noncoding RNAs, such as microRNAs (Konno et al., 2019) and lncRNAs (He et al., 2018), further enhancing the complexity of the epigenetic regulation network.

Although several studies have revealed the role of m6A in the physiopathology of some tumors at the single gene level, a combination of dozens of m6A-related genes may be preferable, especially in prognostic research. In the present study, we observed three m6A readers that were highly expressed in the tumor samples in the TCGA-GTEx cohort and validated by the GEO datasets (Fig. 1). From this perspective, it is difficult to determine whether the overall m6A enrichment is increased in pancreatic tumorigenesis given that both writers and erasers are not differentially expressed, although disruption of the m6A recognition system is likely. In addition, we identified three m6A-related genes that were strongly associated with survival in PAAD. Among these genes, one writer (*RBM15*) and two readers (*HNRNPC* and *IGF2BP2*) were negatively correlated with the survival time and a more dismal prognosis (Fig. 2A). Interestingly, *METTL3* seemed to not be associated with survival in PAAD in this study. A previous study demonstrated that *METTL3* directly associates with the translation machinery and enhances the translation of its target mRNAs (*RGFR* and *TAZ*) independent of its methyltransferase activity in lung cancers. Therefore, in addition to their methyltransferase activity, these m6A writers may impact the prognosis of PAAD via other mechanisms, which explains the unusual observation.

We also investigated the mutation information of the m6A-related genes. Overall, mutations in m6A-related genes were detected in almost 14% of the PAAD samples. The patients whose samples exhibited mutations in m6A-related genes had shorter overall and disease-free survival times than those whose samples did not exhibit mutations. The copy number alteration frequency of *TP53* in the samples with m6A-related mutations was higher than that in the samples without mutations, suggesting that the potential role of m6A modification in pancreatic tumorigenesis is induced by these three driver genes of PAAD. We identified 16 genes that harbored more than 100 m6A loci and showed that a significant association exists between these genes and PAAD survival. In addition, we identified the top 20 genes most strongly associated with PAAD survival. These 36 genes are potential m6A modification targets and, therefore, impact pancreatic oncogenesis.

Few studies have reported the role of m6A-related genes in the immune microenvironment of tumors. Recently, Han et al. uncovered the mechanism by which *YTHDF 1* undermines the durable neoantigen-specific immunity by interacting with transcripts encoding lysosomal proteases. Here, we also found that arm-level deletions and gains of m6A-related genes statistically and significantly decreased the infiltration of CD4+ T cells (*P* < 0.05 and *P* < 0.01, respectively).

This study has some strengths. First, this study is the first to systematically investigate the role of m6A-related genes in the prognosis and immune microenvironment of PAAD using a bioinformatic approach. Generally, more tumor sample data than normal tissue data were recorded in the TCGA database (e.g., only four normal pancreatic tissues are provided), which introduced the risk of bias caused by the “small-sample effect”. Thus, we incorporated the transcriptome data from the GTEx, which collects genetic data exclusively from normal tissues, dramatically enriching our data and enhancing the reliability of our results. Second, a new prognostic model with moderate predictive accuracy based on the expression quantity of three m6A-related genes was constructed in this study and has substantial clinical implications. Third, the genes that harbored the most m6A modification sites were identified from the RMBase database, which is a novel platform created by Sun Yat-sen University that documents plentiful information regarding RNA methylation. We further identified 16 genes associated with PAAD survival that have an abundance of m6A-modified sites, which are potential targets of the m6A-related genes involved in pancreatic tumorigenesis. Finally, this study explored the potential correlation between m6A-related genes and the immune microenvironment of pancreatic cancer for the first time, suggesting that the constitution of infiltrating immune cells may affect the m6A modification of tumor cells. Some limitations should be noted. First, all data were extracted from online databases, and data from biochemical experiments for validation are lacking. Second, although this study indicated the disrupted expression of m6A-related genes, limited knowledge regarding the underlying mechanism involved in pancreatic tumorigenesis was elucidated.

## Conclusion

In conclusion, we observed the differential expression of m6A-related genes between PAAD samples and normal pancreatic tissues, suggesting a novel anticancer strategy for restoring balanced RNA methylation in tumor cells. Future studies are needed to determine the molecular mechanism by which these differentially expressed genes participate in tumorigenesis, metastasis and the immune microenvironment in patients with PAAD.

##  Supplemental Information

10.7717/peerj.9602/supp-1 Figure S1 Flow chart presenting the main design of this study

10.7717/peerj.9602/supp-2 Figure S2 Lasso regression identifies three m6A-related genes for the model construction (A) Curve showing that the partial likelihood deviance changed along with the lambda value. The lambda value is determined when the partial likelihood deviance is at its minimum value. (B) When the lambda value is determined, the corresponding coefficient of each gene can be determined.

10.7717/peerj.9602/supp-3 Figure S3 Validation of the accuracy of the predictive model using a GEO dataset (A) Risk score curve showing that the patients are classified into two groups based on the lasso risk. (B) Survival analysis confirming that the patients with a low lasso risk are characterized by a prolonged overall survival time.

10.7717/peerj.9602/supp-4 Figure S4 Survival analysis of patients with PAAD in terms of sixteen genes among the top 100 genes harboring the most m6A loci

10.7717/peerj.9602/supp-5 Figure S5 Genetic mutation landscape of 21 m6A-related genes

10.7717/peerj.9602/supp-6 Figure S6 Mutation of m6A-related genes affects patients’ prognosis and other gene alterations

10.7717/peerj.9602/supp-7 Figure S7 Consensus clustering analysis dividing the samples into two subgroups (A) The increasing trend of the CDF value with respect to the consensus index was considered indicative of an appropriate classification. (B) The decreasing trend of the relative change in the area under the CDF curve was considered indicative of an appropriate classification.

10.7717/peerj.9602/supp-8 Figure S8 Alteration in YTHDF1 expression in PAAD samples in different T or N stages

10.7717/peerj.9602/supp-9 Table S1 The comparison between pancreatic cancer and normal pancreas in terms of the protein expression of 21 m6A-related genes

10.7717/peerj.9602/supp-10 Table S2 The basic information of the validation cohort from GEO dataset

10.7717/peerj.9602/supp-11 Table S3 The top 100 genes with the most m6A modification number in their transcriptional products

10.7717/peerj.9602/supp-12 Table S4 The number of m6A modification in the transcriptional products of the top 20 genes that are mostly associated with the overall survival of pancreatic cancer

10.7717/peerj.9602/supp-13 Supplemental Information 13 Raw data and corresponding code

## Additional Information and Declarations

### Data Availability

The following information was supplied regarding data availability: Raw data are available at TCGA (search term: TCGA-PAAD). Additional data were collected from NCBI GEO (GSE15471, GSE28735, GSE62452 and GSE11838). The combined data of TCGA-PAAD and GTEx was analyzed using GEPIA (http://gepia.cancer-pku.cn/). All scripts used to analyze the data are available in the Supplemental Files.
