# Supplementary figures and images for: The role of m6A-related genes in the prognosis and immune microenvironment of pancreatic adenocarcinoma
Source: PeerJ. 2020 Sep 28;8:e9602. doi: 10.7717/peerj.9602 (PMC7528816; doi:10.7717/peerj.9602)

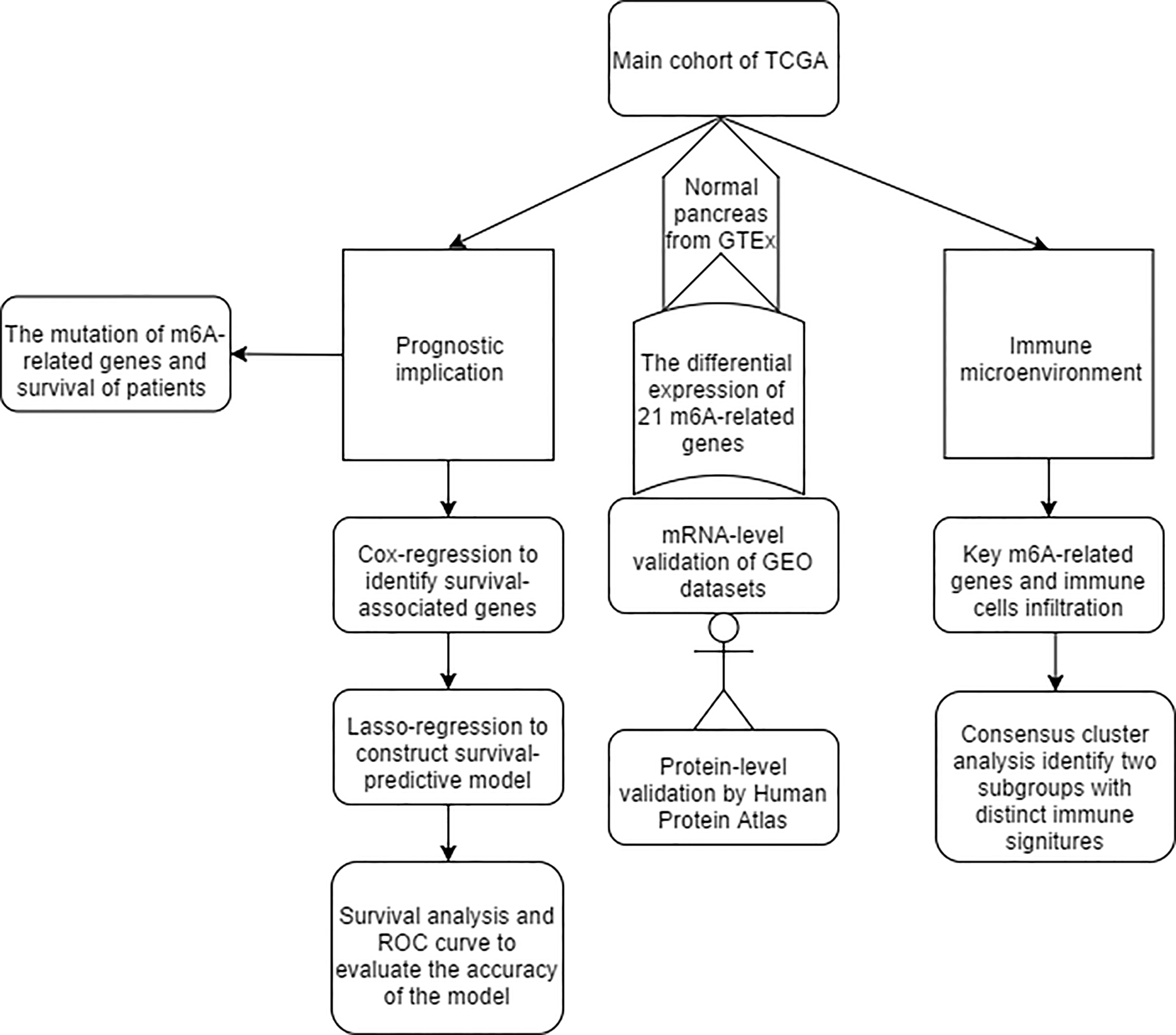

Supplement: Figure S1 [file peerj-08-9602-s001.jpg]

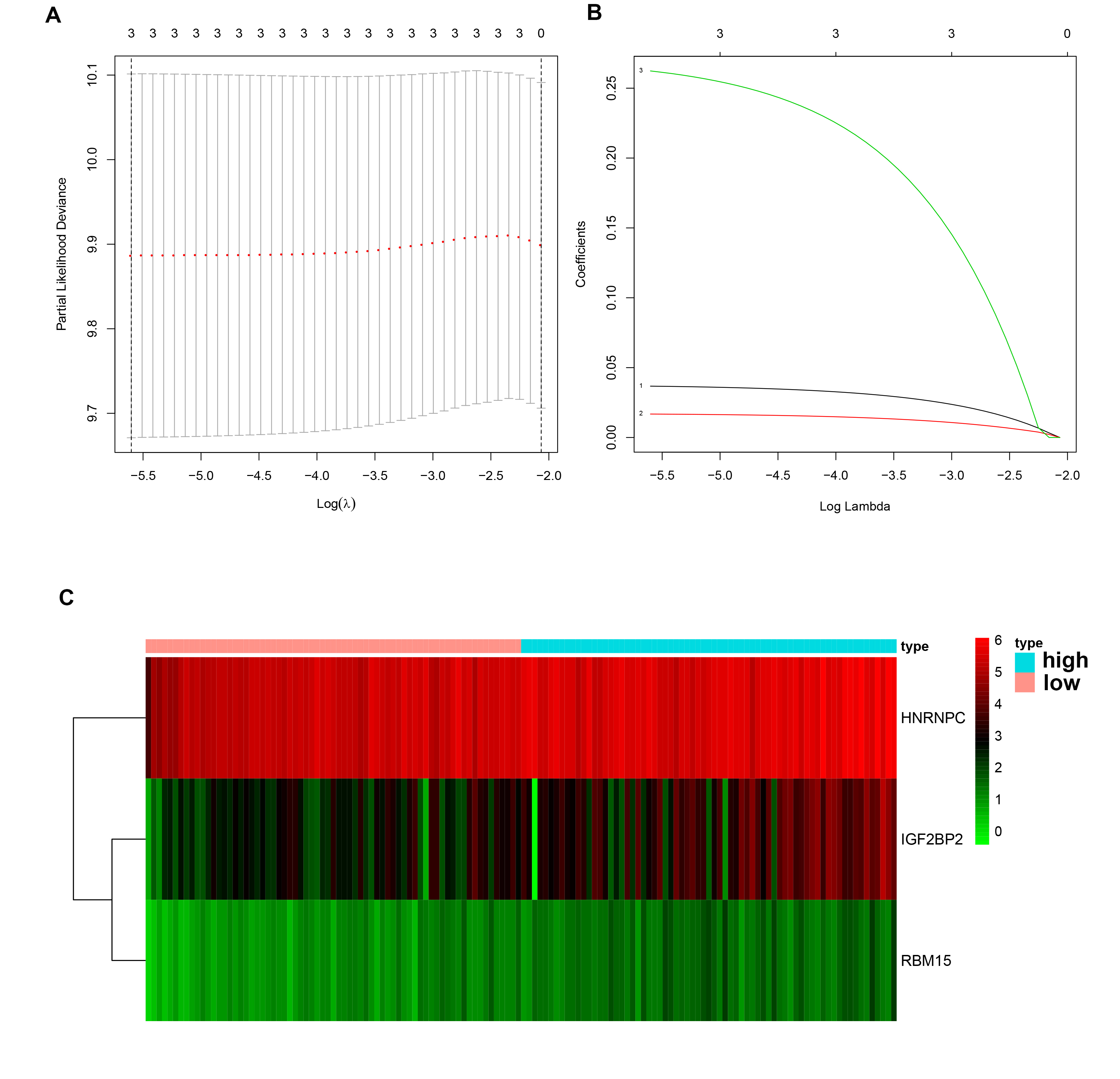

Supplement: Figure S2 — (A) Curve showing that the partial likelihood deviance changed along with the lambda value. The lambda value is determined when the partial likelihood deviance is at its minimum value. (B) When the lambda value is determined, the corresponding coefficient of each gene can be determined. [file peerj-08-9602-s002.jpg]

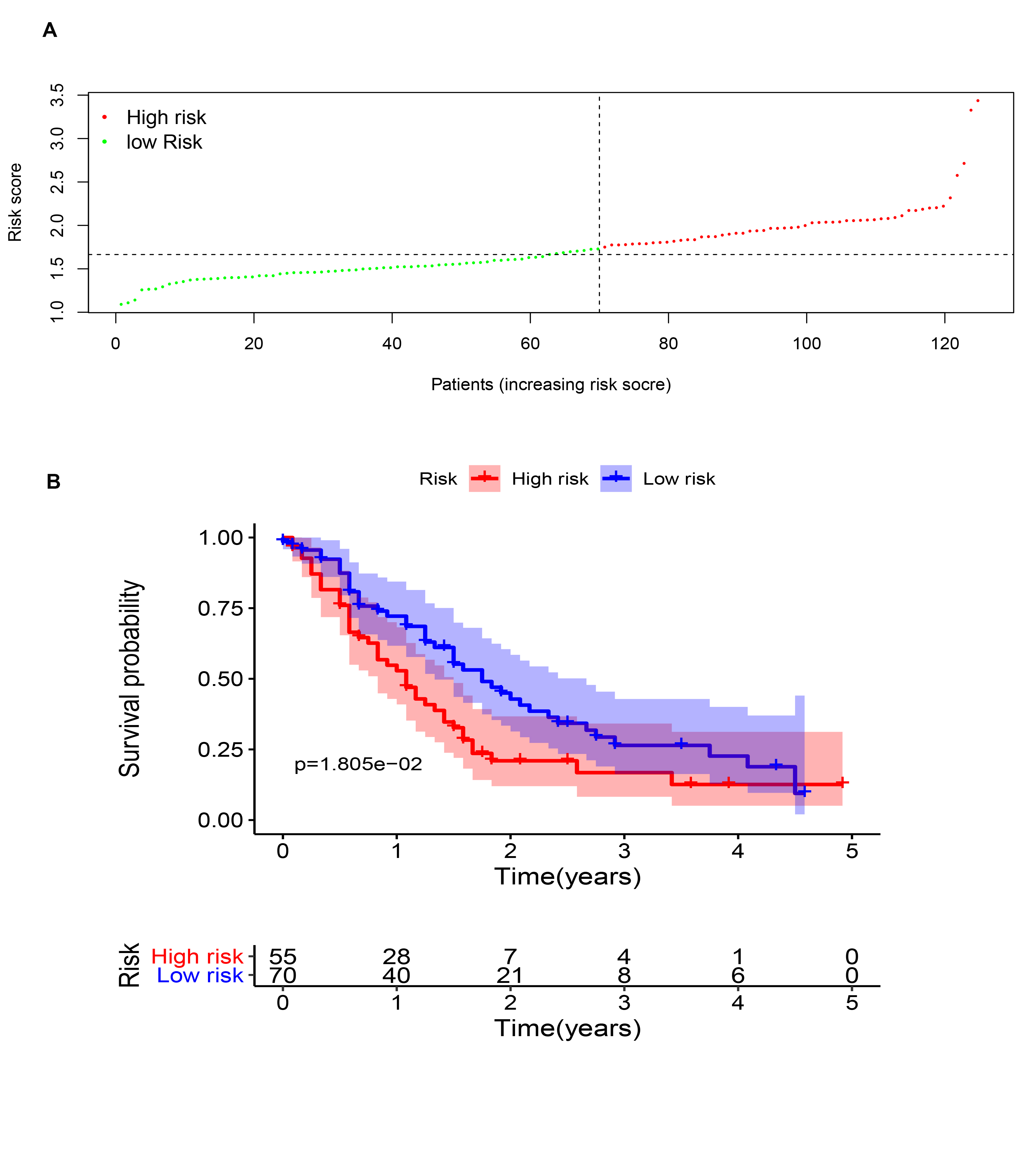

Supplement: Figure S3 — (A) Risk score curve showing that the patients are classified into two groups based on the lasso risk. (B) Survival analysis confirming that the patients with a low lasso risk are characterized by a prolonged overall survival time. [file peerj-08-9602-s003.jpg]

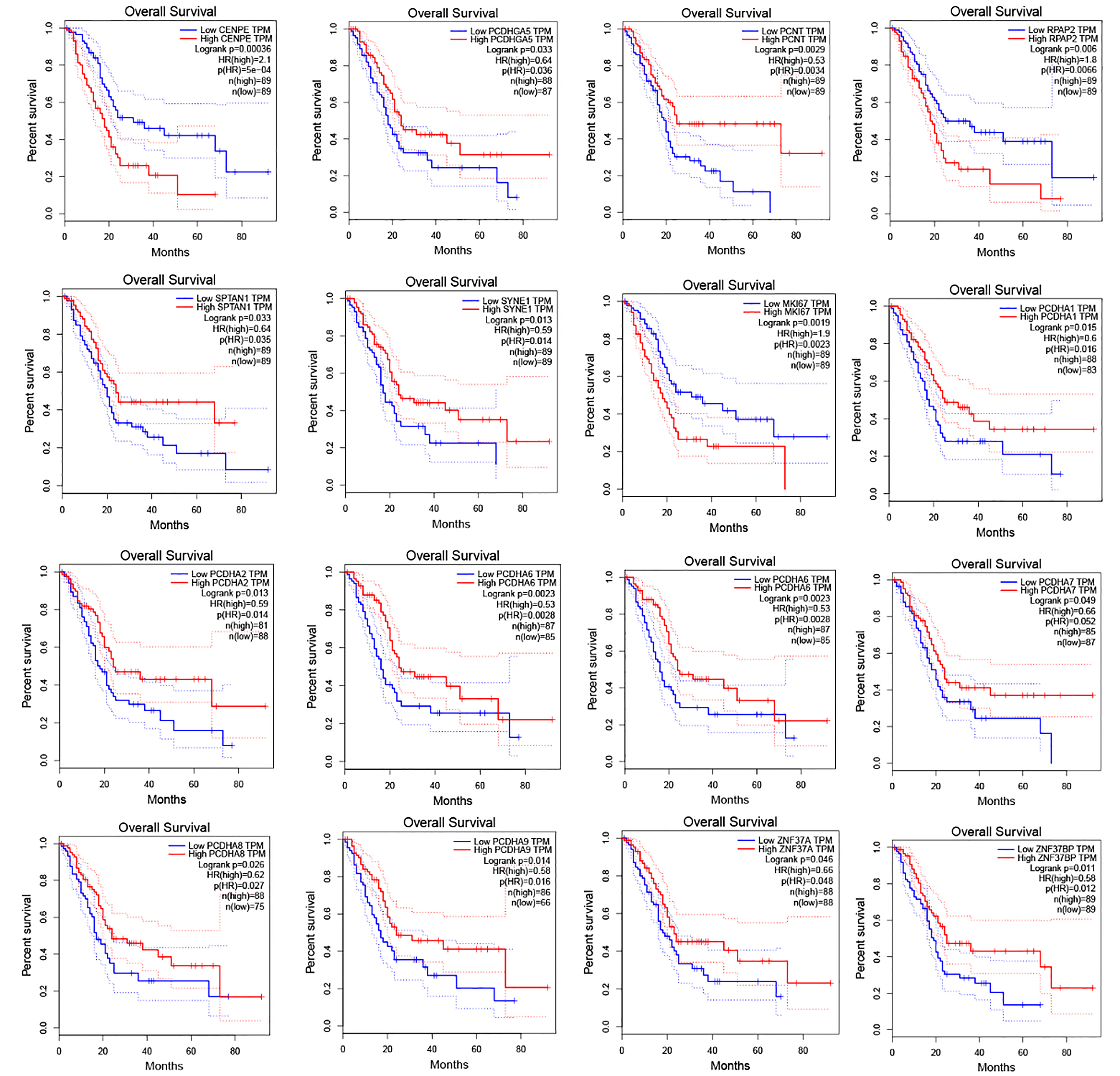

Supplement: Figure S4 [file peerj-08-9602-s004.jpg]

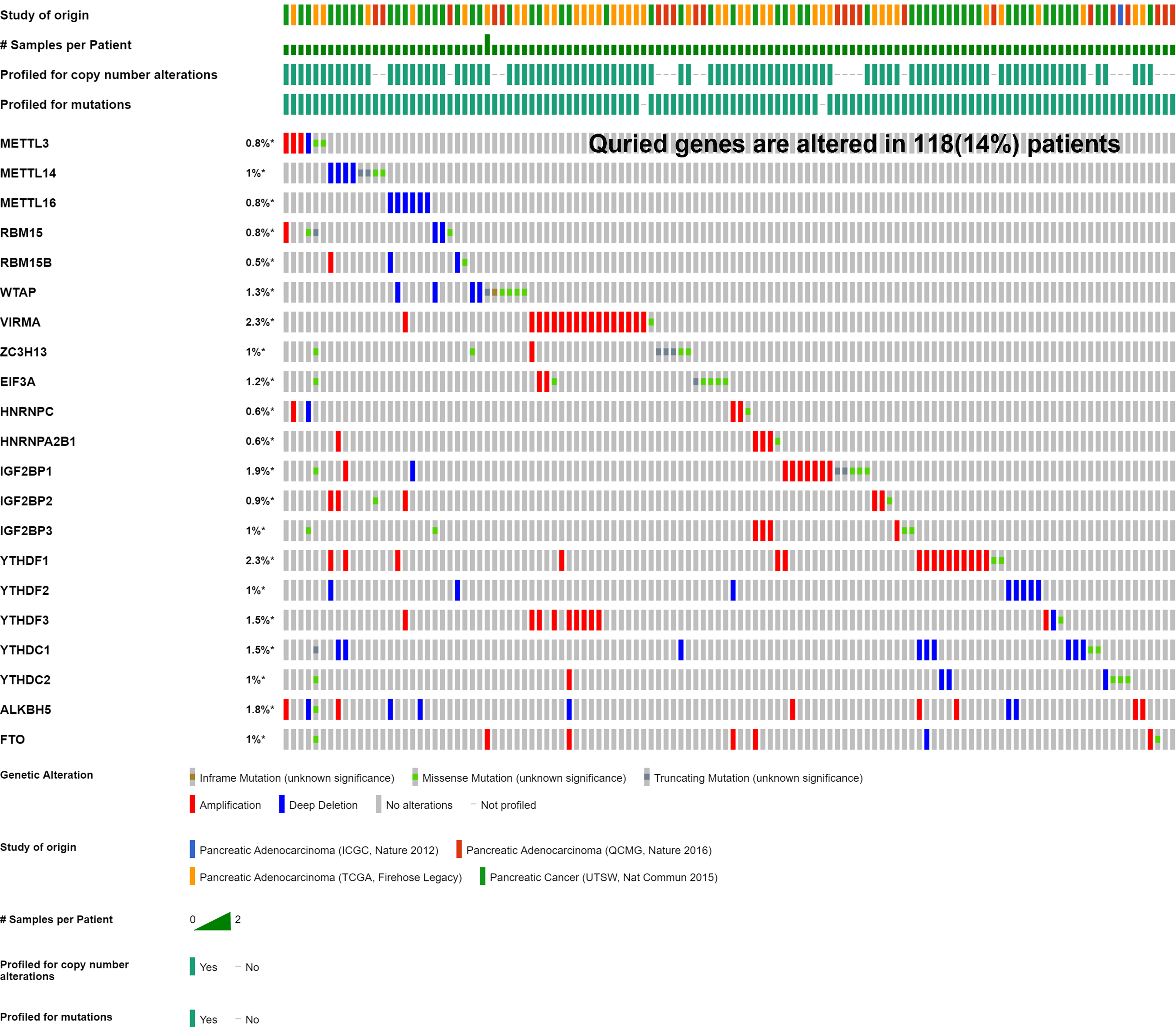

Supplement: Figure S5 [file peerj-08-9602-s005.jpg]

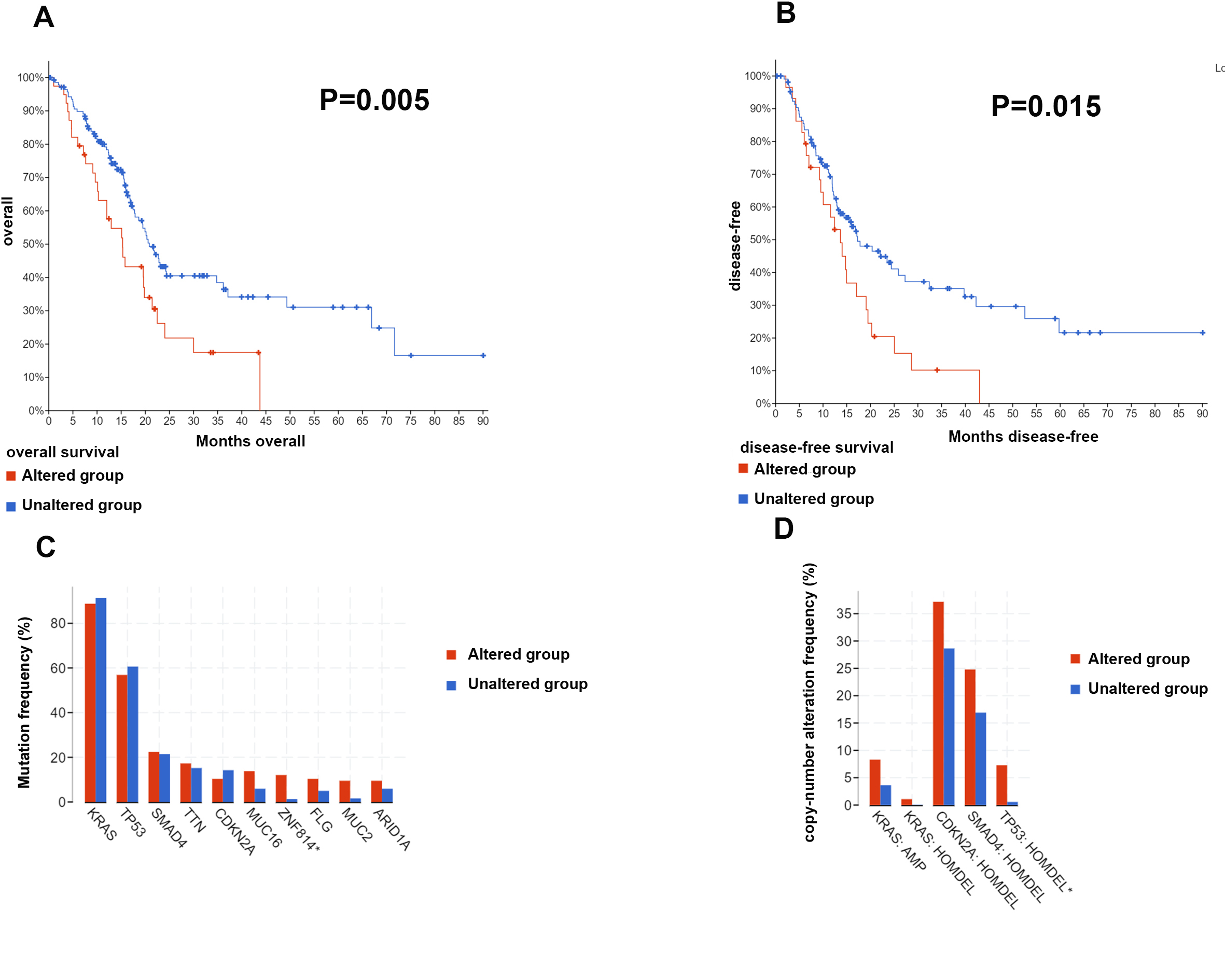

Supplement: Figure S6 [file peerj-08-9602-s006.jpg]

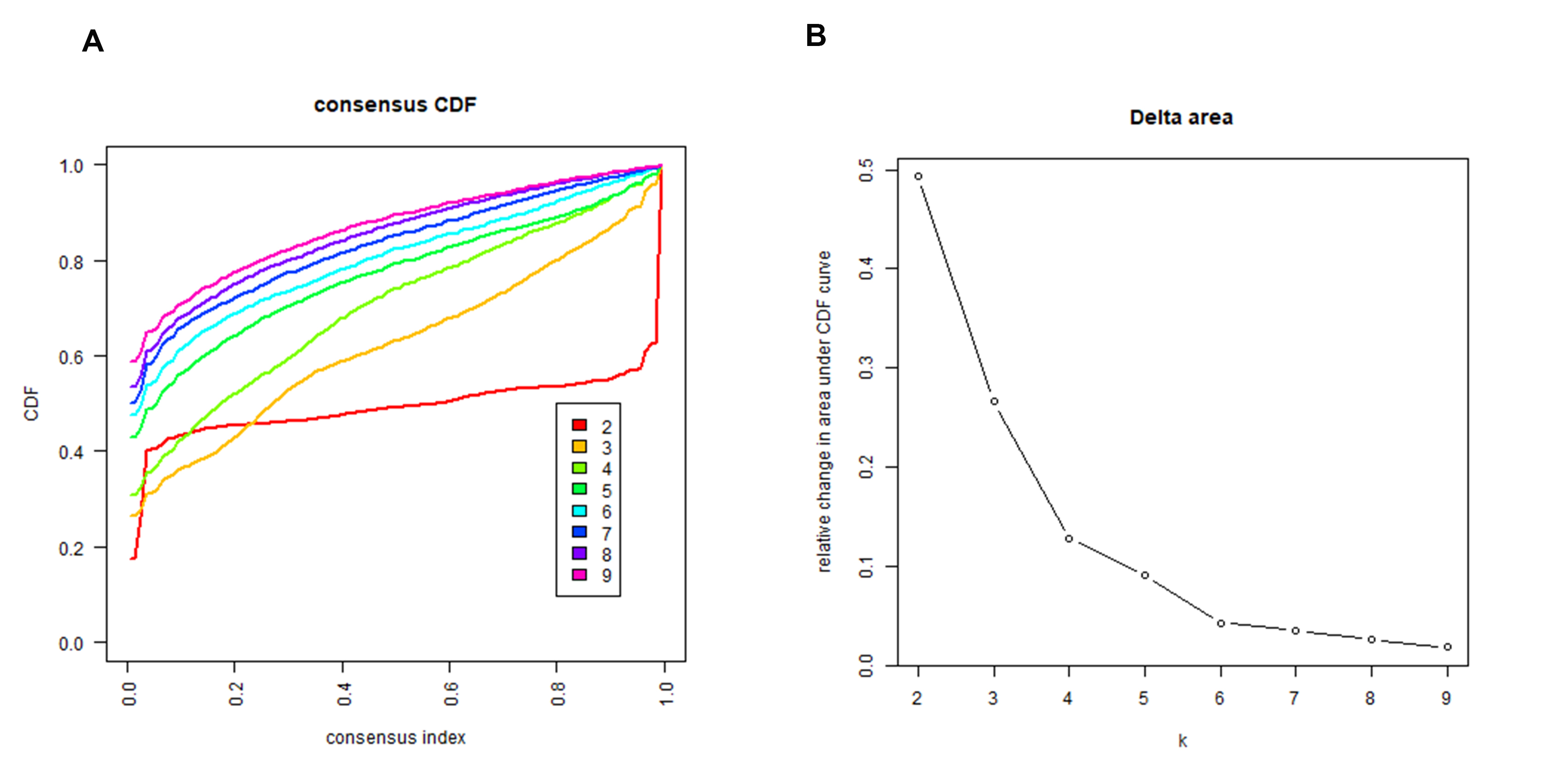

Supplement: Figure S7 — (A) The increasing trend of the CDF value with respect to the consensus index was considered indicative of an appropriate classification. (B) The decreasing trend of the relative change in the area under the CDF curve was considered indicative of an appropriate classification. [file peerj-08-9602-s007.jpg]

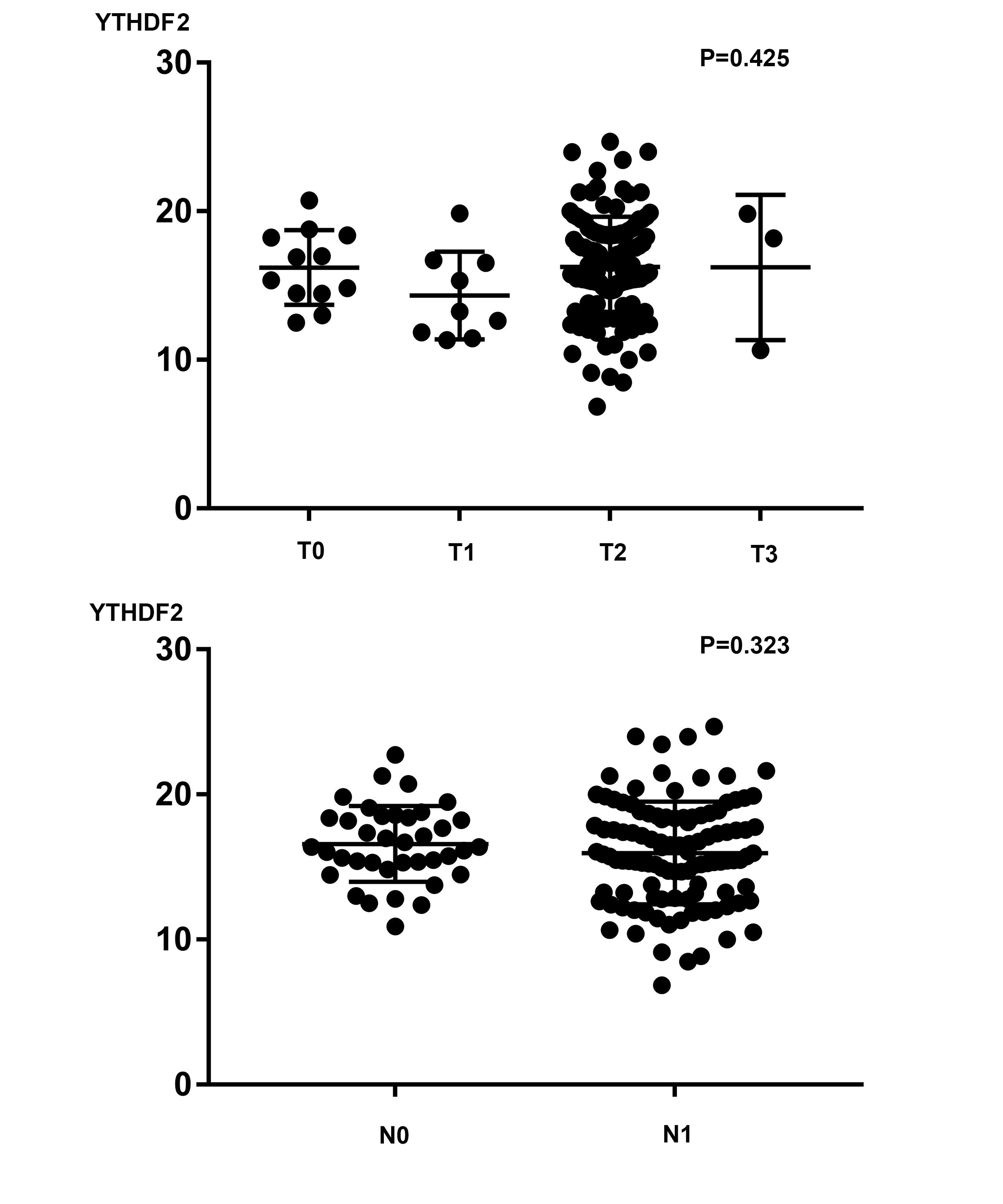

Supplement: Figure S8 [file peerj-08-9602-s008.jpg]
