# Supplementary material for: The role of m6A-related genes in the prognosis and immune microenvironment of pancreatic adenocarcinoma
Source: PeerJ. 2020 Sep 28;8:e9602. doi: 10.7717/peerj.9602 (PMC7528816; doi:10.7717/peerj.9602)
Supplement: Table S1 [file peerj-08-9602-s009.docx]

| Gene_Name | Tumor tissue | | | | Normal tissue | | | |
| --- | --- | --- | --- | --- | --- | --- | --- | --- |
|  | High | Medium | Low | Not detected | High | Medium | Low | Not detected |
| METTL3 |  |  |  |  |  |  |  |  |
| METTL14 | (1/12) | (4/12) | (3/12) | (4/12) |  | 1 |  |  |
| METTL16 |  | (5/10) | (3/10) | (2/10) |  | 1 |  |  |
| RBM15* | (4/11) | (3/11) | (2/11) | (2/11) |  |  | 1 |  |
| RBM15B |  | (1/11) |  | (10/11) |  |  | 1 |  |
| WTAP | (1/11) | (6/11) | (1/11) | (3/11) | 1 |  |  |  |
| KIAA1429 |  | (2/11) | (6/11) | (3/11) |  |  | 1 |  |
| ZC3H13 |  | (6/9) | (2/9) | (1/9) |  | 1 |  |  |
| EIF3A | (3/10) | (6/10) | (1/10) |  | 1 |  |  |  |
| HNRNPC | (10/11） |  | (1/10) |  | 1 |  |  |  |
| HNRNPA2B1 | (7/10) | (3/10) |  |  | 1 |  |  |  |
| IGF2BP1 |  | (1/12) | (4/12) | (7/12) |  |  |  | 1 |
| IGF2BP2 |  | (5/6) | (1/6) |  |  | 1 |  |  |
| IGF2BP3 | (1/12) | (5/12) |  | (6/12) |  |  |  | 1 |
| YTHDF1 |  |  |  |  |  |  |  |  |
| YTHDF2# |  |  | (2/8) | (6/8) | 1 |  |  |  |
| YTHDF3 |  |  |  |  |  |  |  |  |
| YTHDC1 | (8/11) | (3/11) |  |  | 1 |  |  |  |
| YTHDC2* | (5/10) | (4/10) |  | (1/10) |  |  | 1 |  |
| ALKBH5 | (5/12) | (7/12) |  |  | 1 |  |  |  |
| FTO |  | (7/12) | (4/12) | (1/12) |  | 1 |  |  |

**Supplementary table 1.** The comparison between pancreatic cancer and normal pancreas in terms of the protein expression of 21 m6A-related genes.

*. This gene is upregulated in pancreatic cancer; #. This gene is downregulated in pancreatic cancer. No immunohistology data of METTL3, YTHDF1, YTHDF3 is recorded in Human Protein Atlas. The criterial to determine which genes are upregulated in pancreatic cancer: if a specific protein mainly has high and medium staining in pancreatic cancer but low and not detected in normal pancreas tissue, then the gene that encoded this protein is thought to be upregulated in pancreatic cancer.
