# Supplementary material for: The role of m6A-related genes in the prognosis and immune microenvironment of pancreatic adenocarcinoma
Source: PeerJ. 2020 Sep 28;8:e9602. doi: 10.7717/peerj.9602 (PMC7528816; doi:10.7717/peerj.9602)
Supplement: Table S2 [file peerj-08-9602-s010.doc]

**Table S2.** The basic information of the validation cohort from GEO dataset.

| Study | GEO accession | Publication year | Tumor sample | Normal sample |
| --- | --- | --- | --- | --- |
| Whole-Tissue Gene Expression Study of Pancreatic Ductal Adenocarcinoma | GSE15741 | 2009 | 39 | 39 |
| Microarray gene-expression profiles of 45 matching pairs of pancreatic tumor and adjacent non-tumor tissues from 45 patients with pancreatic ductal adenocarcinoma | GSE28735 | 2012 | 45 | 45 |
| Microarray gene-expression profiles of 69 pancreatic tumors and 61 adjacent non-tumor tissue from patients with pancreatic ductal adenocarcinoma | GSE62452 | 2016 | 69 | 69 |
| Pancreatic Tumor vs Various Tissue Normals | GSE11838 | 2008 | 28 | 4 |
| Integrative Survival-Based Molecular Profiling of Human Pancreatic Cancer [mRNA] | GSE32676 | 2011 | 42 | 7 |
| Combinatorial analysis of miRNA and mRNA expression in pancreatic ductal adenocarcinoma (PDAC)_Mrna | GSE41368 | 2013 | 6 | 6 |
| Gene expression of pancreatic tumors | GSE43795 | 2013 | 26 | 5 |
| Molecular analysis of precursor lesions in familial pancreatic cancer | GSE43288 | 2013 | 34 | 6 |
