# Supplementary material for: The role of m6A-related genes in the prognosis and immune microenvironment of pancreatic adenocarcinoma
Source: PeerJ. 2020 Sep 28;8:e9602. doi: 10.7717/peerj.9602 (PMC7528816; doi:10.7717/peerj.9602)
Supplement: Table S3 [file peerj-08-9602-s011.doc]

**Table S3.** The top 100 genes with the most m6A modification number in their transcriptional products.

| Gene | GeneID | Gene Type | Strand | N |
| --- | --- | --- | --- | --- |
| PCDHGA1 | ENSG00000204956.5 | protein_coding | + | 518 |
| PCDHGA2 | ENSG00000081853.14 | protein_coding | + | 503 |
| KCNQ1OT1 | ENSG00000269821.1 | antisense | - | 497 |
| PCDHGB1 | ENSG00000254221.2 | protein_coding | + | 475 |
| PCDHGA4 | ENSG00000262576.2 | protein_coding | + | 453 |
| PCDHGB2 | ENSG00000253910.2 | protein_coding | + | 452 |
| PCDHGA3 | ENSG00000254245.2 | nonsense_mediated_decay,protein_coding | + | 433 |
| PCDHGA5 | ENSG00000253485.2 | protein_coding | + | 428 |
| PCDHGB3 | ENSG00000262209.2 | protein_coding | + | 395 |
| PCDHGA6 | ENSG00000253731.2 | protein_coding | + | 377 |
| PCDHGA7 | ENSG00000253537.2 | protein_coding | + | 365 |
| PCDHGB4 | ENSG00000253953.2 | protein_coding | + | 319 |
| PCDHGA8 | ENSG00000253767.2 | protein_coding | + | 314 |
| PCDHGB5 | ENSG00000276547.1 | protein_coding | + | 298 |
| PCDHA1 | ENSG00000239389.7 | protein_coding | + | 276 |
| FLG | ENSG00000143631.10 | protein_coding | - | 272 |
| PCDHA3 | ENSG00000255408.3 | protein_coding | + | 265 |
| PCDHGA9 | ENSG00000261934.2 | protein_coding | + | 264 |
| PCDHA2 | ENSG00000204969.6 | protein_coding | + | 258 |
| MKI67 | ENSG00000148773.12 | processed_transcript,protein_coding | - | 257 |
| TTN | ENSG00000155657.24 | protein_coding,retained_intron | - | 257 |
| CTD-3105H18.14 | ENSG00000269693.1 | nonsense_mediated_decay | - | 256 |
| PCDHGB6 | ENSG00000253305.2 | protein_coding | + | 243 |
| DST | ENSG00000151914.18 | nonsense_mediated_decay,processed_transcript,protein_coding,retained_intron | - | 240 |
| PCDHA4 | ENSG00000204967.6 | protein_coding | + | 237 |
| MUC16 | ENSG00000181143.15 | nonsense_mediated_decay,processed_transcript,protein_coding | - | 234 |
| PCLO | ENSG00000186472.19 | nonsense_mediated_decay,processed_transcript,protein_coding | - | 233 |
| PCDHA5 | ENSG00000204965.8 | protein_coding | + | 232 |
| AKAP9 | ENSG00000127914.16 | processed_transcript,protein_coding,retained_intron | + | 232 |
| PCDHGA10 | ENSG00000253846.2 | protein_coding | + | 217 |
| PCDHA6 | ENSG00000081842.13 | protein_coding | + | 208 |
| CENPF | ENSG00000117724.12 | processed_transcript,protein_coding | + | 202 |
| RNF213 | ENSG00000173821.19 | processed_transcript,protein_coding,retained_intron | + | 199 |
| GOLGB1 | ENSG00000173230.15 | nonsense_mediated_decay,processed_transcript,protein_coding | - | 196 |
| SPEN | ENSG00000065526.10 | processed_transcript,protein_coding | + | 195 |
| FAT1 | ENSG00000083857.13 | nonsense_mediated_decay,processed_transcript,protein_coding,retained_intron | - | 193 |
| PCDHA7 | ENSG00000204963.4 | protein_coding | + | 190 |
| PCDHGB7 | ENSG00000254122.2 | protein_coding | + | 190 |
| CENPE | ENSG00000138778.11 | processed_transcript,protein_coding,retained_intron | - | 181 |
| PCDHA9 | ENSG00000204961.6 | protein_coding | + | 180 |
| BOD1L1 | ENSG00000038219.12 | nonsense_mediated_decay,protein_coding,retained_intron | - | 178 |
| ALMS1 | ENSG00000116127.17 | nonsense_mediated_decay,processed_transcript,protein_coding,retained_intron | + | 177 |
| PCDHA8 | ENSG00000204962.5 | protein_coding | + | 177 |
| PCDHA10 | ENSG00000250120.6 | protein_coding | + | 177 |
| MAP1A | ENSG00000166963.12 | protein_coding | + | 175 |
| FAT4 | ENSG00000196159.11 | protein_coding,retained_intron | + | 175 |
| KMT2D | ENSG00000167548.14 | processed_transcript,protein_coding,retained_intron | - | 173 |
| APOB | ENSG00000084674.13 | protein_coding | - | 171 |
| MAP1B | ENSG00000131711.14 | nonsense_mediated_decay,processed_transcript,protein_coding | + | 171 |
| PCDHGA11 | ENSG00000253873.5 | protein_coding | + | 170 |
| RPAP2 | ENSG00000122484.8 | processed_transcript,protein_coding,retained_intron | + | 163 |
| KMT2C | ENSG00000055609.17 | nonsense_mediated_decay,processed_transcript,protein_coding,retained_intron | - | 163 |
| ZFHX3 | ENSG00000140836.14 | processed_transcript,protein_coding,retained_intron | - | 162 |
| ZNF469 | ENSG00000225614.2 | protein_coding | + | 157 |
| SPTBN1 | ENSG00000115306.15 | protein_coding,retained_intron | + | 154 |
| PRRC2C | ENSG00000117523.15 | processed_transcript,protein_coding,retained_intron | + | 153 |
| AKAP13 | ENSG00000170776.19 | processed_transcript,protein_coding,retained_intron | + | 149 |
| COL7A1 | ENSG00000114270.15 | processed_transcript,protein_coding,retained_intron | - | 148 |
| ASH1L | ENSG00000116539.10 | nonsense_mediated_decay,protein_coding,retained_intron | - | 147 |
| SYNE2 | ENSG00000054654.15 | nonsense_mediated_decay,processed_transcript,protein_coding,retained_intron | + | 146 |
| STARD9 | ENSG00000159433.11 | nonsense_mediated_decay,protein_coding,retained_intron | + | 141 |
| PEAK1 | ENSG00000173517.10 | processed_transcript,protein_coding,retained_intron | - | 141 |
| BRCA2 | ENSG00000139618.14 | nonsense_mediated_decay,processed_transcript,protein_coding,retained_intron | + | 140 |
| PCDHA11 | ENSG00000249158.6 | protein_coding | + | 140 |
| SETD2 | ENSG00000181555.19 | nonsense_mediated_decay,processed_transcript,protein_coding,retained_intron | - | 138 |
| GVINP1 | ENSG00000254838.5 | processed_transcript,transcribed_unprocessed_pseudogene | - | 137 |
| ZFHX4 | ENSG00000091656.15 | nonsense_mediated_decay,processed_transcript,protein_coding,retained_intron | + | 136 |
| MUC19 | ENSG00000205592.13 | processed_transcript,retained_intron | + | 135 |
| PCDHGA12 | ENSG00000253159.2 | protein_coding | + | 134 |
| ZNF37A | ENSG00000075407.17 | processed_transcript,protein_coding | + | 133 |
| TRIP11 | ENSG00000066427.21 | nonsense_mediated_decay,processed_transcript,protein_coding | - | 133 |
| BPTF | ENSG00000171634.16 | processed_transcript,protein_coding,retained_intron | + | 132 |
| TPR | ENSG00000047410.13 | processed_transcript,protein_coding,retained_intron | - | 131 |
| APC | ENSG00000134982.16 | nonsense_mediated_decay,protein_coding,retained_intron | + | 130 |
| HIVEP2 | ENSG00000010818.8 | processed_transcript,protein_coding | - | 130 |
| CEP350 | ENSG00000135837.15 | nonsense_mediated_decay,processed_transcript,protein_coding | + | 129 |
| ZNF37BP | ENSG00000234420.7 | processed_transcript,transcribed_processed_pseudogene | - | 129 |
| KMT2A | ENSG00000118058.20 | processed_transcript,protein_coding,retained_intron | + | 128 |
| CEP290 | ENSG00000198707.14 | nonsense_mediated_decay,protein_coding | - | 128 |
| TBC1D16 | ENSG00000167291.15 | protein_coding | - | 128 |
| RANBP2 | ENSG00000153201.15 | nonsense_mediated_decay,protein_coding,retained_intron | + | 128 |
| CREBBP | ENSG00000005339.13 | protein_coding,retained_intron | - | 125 |
| TEX15 | ENSG00000133863.6 | processed_transcript,protein_coding | - | 125 |
| FAT3 | ENSG00000165323.15 | processed_transcript,protein_coding,retained_intron | + | 124 |
| ZC3H13 | ENSG00000123200.16 | processed_transcript,protein_coding | - | 124 |
| DIDO1 | ENSG00000101191.16 | protein_coding | - | 124 |
| PCNT | ENSG00000160299.16 | processed_transcript,protein_coding,retained_intron | + | 124 |
| IGF1R | ENSG00000140443.13 | nonsense_mediated_decay,processed_transcript,protein_coding,retained_intron | + | 121 |
| TRIOBP | ENSG00000100106.19 | nonsense_mediated_decay,protein_coding,retained_intron | + | 121 |
| ICE1 | ENSG00000164151.11 | protein_coding,retained_intron | + | 121 |
| EEA1 | ENSG00000102189.16 | nonsense_mediated_decay,processed_transcript,protein_coding | - | 120 |
| TRIP11 | ENSG00000100815.12 | nonsense_mediated_decay,processed_transcript,protein_coding | - | 120 |
| SPTAN1 | ENSG00000197694.13 | processed_transcript,protein_coding,retained_intron | + | 120 |
| NSD1 | ENSG00000165671.18 | nonsense_mediated_decay,protein_coding,retained_intron | + | 119 |
| AKAP12 | ENSG00000131016.16 | processed_transcript,protein_coding | + | 119 |
| SYNE1 | ENSG00000131018.18 | nonsense_mediated_decay,processed_transcript,protein_coding,retained_intron | - | 119 |
| MUC5B | ENSG00000117983.17 | processed_transcript,protein_coding,retained_intron | + | 118 |
| PRR14L | ENSG00000183530.13 | nonsense_mediated_decay,processed_transcript,protein_coding | - | 118 |
| PLEKHG4B | ENSG00000153404.13 | protein_coding,retained_intron | + | 117 |
| BDP1 | ENSG00000145734.18 | nonsense_mediated_decay,protein_coding,retained_intron | + | 116 |

N: The number of m6A modification.
