# Supplementary material for: The role of m6A-related genes in the prognosis and immune microenvironment of pancreatic adenocarcinoma
Source: PeerJ. 2020 Sep 28;8:e9602. doi: 10.7717/peerj.9602 (PMC7528816; doi:10.7717/peerj.9602)
Supplement: Table S4 [file peerj-08-9602-s012.doc]

**Table S4**. The number of m6A modification in the transcriptional products of the top 20 genes that are mostly associated with the overall survival of pancreatic cancer.

| Gene | GeneID | Gene Type | Strand | P | N |
| --- | --- | --- | --- | --- | --- |
| ANKRD19P | ENSG00000187984.12 | processed_transcript,transcribed_unprocessed_pseudogene | + | 1.06E-06 | 19 |
| MYEOV | ENSG00000172927.3 | processed_transcript,protein_coding,retained_intron | + | 1.42E-06 | 10 |
| MMP28 | ENSG00000271447.5 | nonsense_mediated_decay,protein_coding,retained_intron | - | 2.76E-06 | 7 |
| KRT16P3 | ENSG00000214822.8 | \ |  | 4.58E-06 | \ |
| PPEF2 | ENSG00000156194.17 | \ |  | 5.67E-06 | \ |
| ATP6V0E2-AS1 | ENSG00000204934.10 | antisense | - | 6.02E-06 | 32 |
| EFR3B | ENSG00000084710.13 | protein_coding | + | 7.59E-06 | 10 |
| CH17360D5.2 | | ENSG000000276850.4 |  | 8.01E-06 | \ |
| KCNC1 | ENSG00000129159.6 | processed_transcript,protein_coding,retained_intron | + | 8.15E-06 | 18 |
| PLA2G16 | ENSG00000176485.10 | processed_transcript,protein_coding | - | 8.20E-06 | 25 |
| EPS8 | ENSG00000151491.12 | nonsense_mediated_decay,protein_coding,retained_intron | - | 8.57E-06 | 18 |
| CTB-31O20.2 | ENSG00000261526.2 | lincRNA | - | 1.00E-05 | 12 |
| ATP6V1G2-DDX39B | ENSG00000213760.10 | nonsense_mediated_decay,processed_transcript | - | 1.01E-05 | 31 |
| CTA-384D8.35 | ENSG00000272666.1 | lincRNA | - | 1.05E-05 | 6 |
| USP20 | ENSG00000136878.12 | processed_transcript,protein_coding | + | 1.26E-05 | 29 |
| RP11-488C13.5 | ENSG00000258301.3 | lincRNA | - | 1.31E-05 | 22 |
| ANKRD18B | ENSG00000230453.9 | processed_transcript,protein_coding,retained_intron | + | 1.36E-05 | 33 |
| ZNF18 | ENSG00000154957.13 | protein_coding | - | 1.40E-05 | 38 |
| RPSAP52 | ENSG00000241749.4 | processed_transcript,TEC,transcribed_processed_pseudogene | - | 1.61E-05 | 5 |
| AC025165.8 | ENSG00000224713.4 | antisense | - | 1.64E-05 | 11 |

P: The p value reflected the relevance between the identified genes and the overall survival of pancreatic cancer. N: The number of m6A modification in the transcriptional products of these identified genes.
